# Cost-effectiveness of total shoulder arthroplasty: A systematic review of the evidence

**DOI:** 10.1007/s00264-026-06830-2

**Published:** 2026-06-29

**Authors:** Stephen D Gill, Shayma Mohammed Selim, Richard S Page, Kevin Eng, Nadine E Foster, Mary Lou Chatterton

**Affiliations:** 1 School of Medicine, Deakin University, Waurn Ponds, Australia; 2 Orthopaedic Department, Barwon Health, Geelong, Australia; 3 Evaluation Office, Centre for Healthcare Innovation, NHG Health, Singapore, Singapore; 4 STARS Education and Research Alliance, Surgical Treatment and Rehabilitation Service (STARS), The University of Queensland and Metro North Health, Brisbane, Australia; 5 School of Public Health and Preventive Medicine, Monash University, Melbourne, Australia; 6 Barwon Centre for Orthopaedic Research and Education (B-CORE), St John of God Hospital, Geelong, Australia; 7 Centre for Innovation in Pain and Health Research, School of Health and Rehabilitation Sciences, The University of Queensland, Brisbane, Australia

**Keywords:** Shoulder, Arthroplasty, Cost-effectiveness, Economic analysis, Systematic review, Total shoulder replacement

## Abstract

**Background:**

Total shoulder arthroplasty (TSA) is increasingly used to treat a range of shoulder disorders. Although TSA improves pain and function, its cost-effectiveness remains uncertain. This systematic review evaluated the cost-effectiveness of TSA for glenohumeral arthritis, rotator cuff arthropathy, massive rotator cuff tears and proximal humerus fractures.

**Methods:**

We systematically searched MEDLINE, Embase, and CINAHL from inception to July 2025 for full economic evaluations comparing TSA with surgical or non-operative alternatives. Eligible studies included primary anatomical total shoulder arthroplasty (ATSA) or reverse total shoulder arthroplasty (RTSA). Studies could be randomised, observational or model-based, and had to provide costs and outcomes to allow determination of an incremental cost-effectiveness ratio or equivalent expression. Study quality was assessed with the Drummond checklist. Given study heterogeneity, results were synthesised narratively.

**Results:**

Of 308 potentially eligible studies, 26 were included, comprising 15,163 patients, all of which were conducted in high-income countries. Fifteen studies were modelled, relying on published literature for costs and outcomes. Most studies were rated as fair (58%) or good (38%) quality. In four studies regarding osteoarthritis, ATSA was consistently cost-effective or dominant (more effective and less costly) over hemiarthroplasty. In three studies regarding osteoarthritis with intact rotator cuff, ATSA and RTSA were similarly effective, and costs were similar or less for ATSA, depending on patient age. In four studies regarding proximal humerus fractures, RTSA was cost-effective or dominant over hemiarthroplasty. In three studies regarding massive rotator cuff tears, cuff repair was always substantially less costly and marginally more effective than RTSA.

**Conclusions:**

Despite the increasing use of TSA, evidence regarding its cost-effectiveness is limited to a small number of studies from high-income countries. Although TSA might be cost-effective for selected indications, high-quality, randomised, prospective economic evaluations from a diverse range of settings are needed to provide rigorous evidence to guide value-based care in shoulder arthroplasty.

**Supplementary Information:**

The online version contains supplementary material available at https://doi.org/10.1007/s00264-026-06830-2.

## Introduction

Shoulder arthroplasty improves pain and function in people with advanced glenohumeral arthritis, rotator cuff arthropathy, massive rotator cuff tears or proximal humeral fractures [1]. The incidence of shoulder arthroplasty has risen exponentially worldwide over the last two decades [1], likely driven by expanding indications and the introduction of reverse total shoulder arthroplasty (RTSA) [1]. RTSA was developed to improve outcomes in rotator cuff-deficient patients and is now also used in patients with primary osteoarthritis (OA) and fractures [4, 5]. RTSA has overtaken anatomical total shoulder arthroplasty (ATSA) to become the most common shoulder arthroplasty in many countries [2, 6, 7]. Conversely, the use of hemi-arthroplasty (HA) is declining in favour of total shoulder arthroplasty (TSA), particularly RTSA [1, 2, 6].

Clinically, patients usually report reduced pain and improved function following TSA [2]. However, there is no *high-quality*evidence from randomised controlled trials (RCTs) demonstrating TSA’s superiority over other treatments [8]. Beyond clinical effectiveness, economic evaluations are necessary to inform value-based care. Full economic evaluations compare costs and outcomes for two or more treatments and differ according to the method for measuring outcomes. Cost-effectiveness analysis uses clinically meaningful health outcomes such as pain; cost-utility analysis uses quality-adjusted life-years (QALYs), which consider quality and length of life; and cost–benefit analysis expresses outcomes in monetary units. Results of cost-effectiveness analyses and cost-utility analyses are reported as incremental cost-effectiveness ratios (ICER), whereas cost–benefit analyses are reported as net monetary benefit.

Despite the increase in TSAs, few economic evaluations have been conducted to date. A systematic review in 2021 by Cregar et al. identified nine studies. [9] They reported that most studies were good quality, with RTSA being cost-effective compared to non-operative treatment and HA, while ATSA was cost-effective versus HA. However, the review was limited to studies in the USA. Additional studies have since been published, warranting an updated systematic review.

The aim of this systematic review was to identify and synthesise health economic evaluations of TSA for glenohumeral arthritis, rotator cuff arthropathy, massive rotator cuff tears, and proximal humerus fracture to assess TSA’s value for money relative to other surgical or non-surgical options.

## Methods

The study was conducted in alignment with PRISMA guidelines for systematic reviews and meta-analyses [10] and registered with PROSPERO (CRD42021237270).

### Study selection criteria and eligibility

Eligible studies included patients undergoing primary TSA to treat glenohumeral arthritis, rotator cuff arthropathy, massive rotator cuff tear without arthropathy, or proximal humeral fracture, regardless of prosthesis type or patient age. Studies had to compare ATSA and/or RTSA with another shoulder intervention (e.g. alternative surgical technique and/or non-operative care) and provide cost-outcome analyses allowing calculation of an ICER or equivalent expression. Studies could include randomised controlled trials (RCTs) and observational studies, or could use simulated data generated through economic modelling. To ensure comprehensiveness of the review, there were no restrictions on study design, sample size or study country. Articles in languages other than English, conference abstracts, revision arthroplasty, partial economic evaluations reporting only costs or outcomes, and studies that did not compare TSA to another group or shoulder intervention were excluded.

### Information sources and search strategy

Medline, Embase and CINAHL databases were searched from inception until 28 July 2025. Search terms were developed using the concepts “shoulder arthroplasty/replacement” and “cost”. Search strategies are included as a supplementary appendix (Supplementary Table 1). Results were exported into Covidence for screening. Reference lists were reviewed for additional studies. Two reviewers (MLC, SMS) independently screened titles and abstracts, with full-text review conducted when necessary. A third reviewer (SDG) arbitrated disagreements.

### Data extraction

Data were independently extracted by two reviewers (MLC, SMS) using predefined templates. Inconsistencies were arbitrated by a third reviewer (SDG). Extracted data included study design, patient characteristics, interventions and outcomes. Costs were converted to USD, the most commonly reported currency in the included studies (Table 1) [11]. Original costs were inflation-adjusted to 2024 values using country-specific health-related Consumer Price Index (CPI) data [12] and then converted to USD via 2024 Purchasing Power Parity data [13]. When year of pricing was missing, it was assumed as publication year.

**Table 1:** Study characteristics

Study (year), Country	Study design	Evaluation type	Population/ reference case (and sample size where relevant)	Preoperative pathology	Interventions and comparators	Perspective, Time horizon	Year of pricing, discount rates, currency	Costs (converted to USD, 2024)	Outcome (QALY, PVI or natural units)	Cost per unit difference in outcome (converted to USD, 2024); Original authors’ conclusions
OSTEOARTHRITIS, ROTATOR CUFF ARTHROPATHY OR MIXED DIAGNOSIS										
Berglund et al. (A) (2019), USA [5]	Retrospective analysis of institutional data	Procedure value index (natural units, PROMS). Mean improvement expressed in units of MCID	Patients undergoing primary TSA between 2007—2015 at a single institution, n = 534	Mixed diagnosis: OA (63%), RCA (36%), inflammatory arthritis (3%), AVN (2%), old fracture (4%), dislocation (1%), RCT (1%) (more than 1 diagnosis possible)	1. ATSA (95.9% cuff intact) 2. RTSA (87.9% cuff tear)	Payer (Medicare and private insurance) and hospital cost perspectives with a minimum 2 year follow up	Data collected between 2007—2015, discounting not stated, USD	ATSA v RTSA: $13,281 v $14,865 ("total hospital cost")	ATSA v RTSA: SST score 1.65 v 1.52 (p. = 159) ASES score 1.72 v 1.49 (p =.010) VAS pain score 1.66 v 1.50 (p =.105) SANE score 1.09 v 1.23 (p. = 150)	SST score: -$12,184 (Dominant) ASES score: -$6,887 (Dominant) VAS pain score: -$9,899 (Dominant) SANE score: $11,314: ATSA cheaper with similar outcomes
Berglund et al. (B) (2019), USA [6]	Retrospective analysis of institutional data	Procedure value index (natural units, PROMS) Mean improvement expressed in units of MCID	Patients undergoing primary RTSA from 2007—2015 at a single institution, n = 176	Mixed diagnosis: RCA (87%), OA (14%), fracture (18%), failed cuff repair (7%), AVN (4%), inflammatory arthritis (3%), dislocation (3%), RCT (2%), (more than 1 diagnosis possible)	1. RTSA cemented 2. RTSA press-fit humeral stem	Hospital cost perspective with a minimum 2 year follow up	Data collected between 2007—2015, discounting not stated, USD	RTSA cemented v RTSA press-fit: $17,043 v $12,952(p =.001)	RTSA cemented v RTSA press-fit SST change score: 5.12 v 5.73 (p =.23)	-$7,297 (Dominated); RTSA had higher costs and similar outcomes
Bhat et al. (2016), USA [7]	Modelled	Cost-utility analysis (QALY)	30—50 year old patients with glenohumeral OA	OA	1. ATSA 2. HA	Health system payer perspective (Medicare reimbursement) for a patient's lifetime	2014, discounting not stated, USD	ATSA v HA: $30,536 v $32,211 (reimbursements per patient)	ATSA v HA: 7.96 v 6.55	-$1,188 (Dominant); ATSA “cost-effective”
Chawla et al. (2021), USA [12]	Retrospective analysis of institutional data	Cost and outcome description	Patients undergoing shoulder arthroplasty between 2011—2016 at a single institution, n = 433	Mixed diagnosis: OA (80%), capsulorrhaphy arthropathy (8%), other (12%)	1. ATSA 2. Ream-and-run arthroplasty (R&R)	Hospital costs perspective with 2 year follow up	No single year of pricing was noted, 3% annual discount rate, USD	ATSA v R&R: $12,717 v $10,637 (p <.001)	ATSA v R&R: 1.05 vs 1.19 (p =.361)	-$14,856 (Dominated); ATSA higher costs and similar outcomes
Chawla et al. (2022), USA [11]	Retrospective analysis of institutional data	Cost and outcome description using QALYs	Patients undergoing RTSA or HA for RCA between 2010—2016 at a single institution, n = 72	RCA	1. RTSA 2. HA	Hospital costs with 2 year follow up	No single year of pricing was noted, 3% annual discount rate, USD	RTSA v HA: $16,025 v $11,044 (p <.001)	RTSA v HA:.75 v.79 (p =.90)	-$124,511 (Dominated); RTSA higher costs and similar outcomes
Coe et al. (2012), USA [13]	Retrospective cohort analysis & Markov modelling of costs	Cost-utility analysis (QALY)	70 year old patient of average health requiring surgery for RCA, n = 42	RCA	1. RTSA 2. HA	Health system payer perspective (Medicare reimbursement and implant prices) for a patient's lifetime	2008—9, 3% annual discount rate, USD	RTSA v HA: $33,705 v $17,585	RTSA v HA: 6.45 v 6.33	$134,331; RTSA is cost-effective^c^
Davies et al. (2025), UK [18]	Modelled	Cost-utility analysis (QALY)	Patients with glenohumeral OA with an intact rotator cuff	OA with intact cuff	1. ATSA 2. HA	Hospital costs over a patient's lifetime	2022/2023, 3.5% discount rate applied for costs and health outcomes, GBP	Female ≤ 60 ATSA v HA: $13,663 v $14,639 Male ≤ 60 ATSA v HA: $12,755 v $13,441 Female 61—75 ATSA v HA: $11,182 v $10,913 Male 61—75 ATSA v HA: $10,801 v $10,214 Female > 75 ATSA v HA: $10,164 v $8,733 Male > 75 ATSA v HA: $10,084 v $8,743	Female ≤ 60 ATSA v HA: 13.63 v 11.64 Male ≤ 60 ATSA v HA: 13.01 v 11.00 Female 61—75 ATSA v HA: 9.30 v 7.85 Male 61—75 ATSA v HA: 8.51 v 7.25 Female > 75 ATSA v HA: 5.26 v 4.32 Male > 75 ATSA v HA: 4.60 v 3.87	Female ≤ 60: -$491 (Dominant) Male ≤ 60: -$341 (Dominant) Female 61—75: $185 Male 61—75: $466 Female > 75: $1,522 Male > 75: $1,837; ATSA dominant in young people and cost-effective for ≥ 61 years of age
Flinkkila et al. (2024), Finland [22]	Retrospective cohort analysis & Markov modelling of costs	Cost-utility analysis (QALY)	Patients with glenohumeral OA or RCA with massive irreparable rotator cuff tear, n = 260	OA or RCA	1. ATSA (OA) 2. RTSA (RCA)	Publicly funded health care system perspective (Finland) for a patient's lifetime	No single year of pricing was noted, no discounting in primary model, Euro	ATSA v RTSA: $14,138 v $15,616	ATSA v RTSA:.54 v.32 (95% CI 0.10—0.54)	-$6,719 (Dominant); "RTSA is less cost-effective"
Grobet et al. (2021), Switzerland [25]	Prospective analysis	Cost-utility analysis (QALY)	Patients undergoing TSA between 2014—2016 at a single institution, n = 150	Mixed diagnosis: OA (46%), RCA (40%), RCT (11%), old fracture (3%)	1. ATSA (31%) or RTSA (69%) 2. Nonoperative management	Health care system and societal (productivity) perspectives with 2 year follow up	2018, no discounting, Swiss francs	TSA v nonoperative: $57,835 v $35,351 (non-working patient) TSA v nonoperative: $119,158 v $149,938 (working patient)^a^	TSA v nonoperative: 1.73 v 1.36 (at 2 years)	Non-working patient: $60,770 Working patient: -$69,674; TSA is cost effective and dominant for the working patient
Lapner et al. (2021), Canada [28]	Modelled	Cost-utility analysis (QALY)	Data from a retrospective cohort study comparing 10-year revision risk for TSA and HA among 5777 patients between 2002—2012 for glenohumeral arthritis	OA and RA	1. ATSA 2. HA	Publicly funded health care system perspective (Canada) for a patient's lifetime	2019, 1.5% annual discount rate, USD	ATSA v HA: $115,785 v $118,501	ATSA v HA: 10.21 v 8.47	-$1,783 (Dominant); ATSA dominant
Mather et al. (2010), USA [30]	Modelled	Cost-utility analysis (QALY)	64 year old patients with glenohumeral OA	OA	1. ATSA (assumed anatomical) 2. HA	Health system payer perspective (Medicare reimbursement) for a patient's lifetime	2008, costs and utilities discounted at 3%, USD	ATSA v HA: $17,109 v $19,996	ATSA v HA: 12.19 v 11.43	-$3,799 (Dominant); ATSA dominant
Oeding et al. (2024), USA [39]	Modelled	Cost-utility analysis (QALY)	72 year old patient with end-stage glenohumeral OA	OA with intact cuff	1. ATSA 2. RTSA	Hospital perspective (institutional cost data) for a patient’s lifetime	No single year of pricing was noted, 3% annual discount rate to costs and outcomes, USD	ATSA v RTSA: $29,077 v $31,199	ATSA v RTSA: 9.43 v 9.44	$212,200; ATSA cost-effective^b^ (in adults for > 67 years of age)
Polisetty et al. (2021), USA [42]	Retrospective analysis of institutional data	Procedure value index (natural units, PROMS) Mean improvement expressed in units of MCID	Patients undergoing TSA between 2007—2018 at a single institution, n = 315	OA with intact cuff	1. ATSA 2. RTSA	Hospital, patient (charges billed to patient by hospital) and payer (Medicare and private insurance reimbursement) perspectives with minimum 2 year follow up	Data collected between 2007—2018, discounting not stated, USD	ATSA v RTSA: $10,851 v $11,360 ("total cost") (p =.116)	ATSA v RTSA: SST score 1.97 v 2.06 (p =.402) ASES score: 1.64 v 1.81 (p =.125) VAS pain score: 1.08 v 1.15 (p =.456) SANE score: 1.42 v 1.31 (p =.492)	SST score: $5,650 ASES score: $2,991 VAS pain score: $7,264 SANE score: -$4,623 (Dominant): ATSA and RTSA demonstrated similar costs and outcomes
Valsamis et al. (2024), UK [50]	Retrospective cohort analysis & Markov modelling of costs	Cost effectiveness analysis (natural units, PROMS)	Patients with glenohumeral OA and intact rotator cuff aged 60 years and older, n = 7124	OA with intact cuff	1. ATSA 2. RTSA	Health system payer perspective (institutional cost data) for a patient’s lifetime	2020/21, 3.5% annual discount rate, GBP	ATSA v RTSA: $17,112 v $17,346 (primary) (p >.05)	ATSA v RTSA: 21.8 v 21.6 (p >.05)	-$1,167 (Dominant); no statistically significant and clinically important differences in outcomes or costs
PROXIMAL HUMERUS FRACTURES										
Abdel Khalik et al. (2022), Canada [1]	Modelled	Cost-utility analysis (QALY)	75 year old patient with three or four-part proximal humerus fracture	Fracture, proximal humerus	1. RTSA 2. ORIF 3. HA 4. Nonoperative management	Single-payer governmental (Canada) healthcare perspective for a patient's lifetime	2020, 3% annual discount rate for costs and outcomes, Canadian $	RTSA v ORIF: $24,079 v $22, 313 RTSA v HA: $24,079 v $23,093 RTSA v nonoperative: $24,079 v $18,177	RTSA v ORIF:7.92 v 6.51 RTSA v HA: 7.92 v 7.31 RTSA v nonoperative: 7.92 v 7.12	RTSA v ORIF: $1,253 RTSA v HA: $1,616 RTSA v nonoperative: $7,377; RTSA cost effective
Austin et al. (2020), USA [3]	Modelled	Cost-utility analysis (QALY)	75 year old patient with proximal humerus fracture requiring surgery and Charlson comorbidity score of 1	Fracture, proximal humerus	1. RTSA 2. ORIF	Hospital charges and payer (Medicare) perspectives for a patient's lifetime	2018, 3% annual discount rate, USD	RTSA v ORIF: $20,755 v $16,784 (payer perspective) RTSA v ORIF: $114,061 v $86,479 (hospital perspective)	RTSA v ORIF: 7.11 v 6.22	Payer perspective $4,462 Hospital perspective $30,991; RTSA cost effective
Bjordal et al. (2022), Norway [8]	Within trial	Cost-utility analysis (QALY)	65—85 years old with displaced proximal humerus fractures who required surgery were randomly allocated to RTSA or ORIF, n = 124	Fracture, proximal humerus	1. RTSA 2. ORIF	Health care perspective (Norway) with 2 year follow up	2016, 4% annual discount rate, Euros	RTSA v ORIF: $58,285 v $50,670	RTSA V ORIF: 1.24 v 1.26	-$380,756 (Dominated); RTSA dominated^d^
Nwachukwu et al. (2016), USA [36]	Modelled	Cost-utility analysis (QALY)	70 year old patient with complex proximal humerus fracture	Fracture, proximal humerus	1. RTSA 2. HA 3. Nonoperative management	Hospital cost and payer (Medicare reimbursement) perspectives for a patient’s lifetime	2013, 3% annual discount rate, USD	1. RTSA v HA: $23,960 v $20,253 (payer perspective) 2. RTSA v HA: $122,049 v $84,190 (hospital perspective) 3. RTSA v nonoperative: $23,960 v $0 (payer perspective) 4. RTSA v nonoperative: $122,975 v $0 (hospital perspective)	1. RTSA v HA: 10.18 v 9.64 2. RTSA v nonoperative: 10.18 v 7.91	RTSA v HA (payer perspective) $6,864 RTSA v HA (hospital perspective) $70,109 RTSA v nonoperative (payer perspective) $10,555 RTSA v nonoperative (hospital perspective) $54,174; RTSA cost effective
Osterhoff et al. (2017), Canada [40]	Modelled	Cost-utility analysis (QALY)	72 year old female patient with complex proximal humerus fracture	Fracture, proximal humerus	1. RTSA 2. HA	Single-payer governmental (Canadian provincial government—Ontario Ministry of Health) healthcare perspective for a patient’s lifetime	2014, 5% annual discount rate, Canadian $	RTSA v HA: $25,558 v $19,362	RTSA v HA: 6.19 v 5.76	$14,409; RTSA cost effective
Sheibani-Rad et al. (2021), USA [46]	Modelled	Cost-utility analysis (QALY)	75 year old patient with proximal humerus fracture requiring surgery	Fracture, proximal humerus	1. RTSA 2. HA	Healthcare payer perspective for a patient's lifetime	2019, 3% discount rate, USD	RTSA v HA: $59,559 v $60,698	RTSA v HA: 6.62 v 6.45	-$6,700 (Dominant); RTSA cost effective
ROTATOR CUFF TEAR										
Castagna et al. (2019), Italy [10]	Modelled	Cost-utility analysis (QALY)	Patients with irreparable rotator cuff tear (RCA or glenohumeral arthritis not mentioned)	RCT	1. RTSA 2. Rotator cuff repair 3. Subacromial Spacer (comparison group) 4. Nonoperative management	Health system payer perspective (Medicare reimbursement) for 24 months	2017, cumulative inflation based on CPI, discounting not stated, Euros	RTSA v nonoperative: $52,427 v $28,392 RTSA v spacer $52,427 v $29,274 RTSA v cuff repair $54,427 v $41,075	RTSA v nonoperative: 1.28 v 1.33 RTSA v spacer: 1.28 v 1.38 RTSA v cuff repair: 1.28 v 1.32	RTSA v nonoperative: -$480,698 (Dominated) RTSA v spacer: -$231,530 (Dominated) RTSA v cuff repair: -$283,795 (Dominated); RTSA dominated
Dornan et al. (2017), USA [19]	Modelled	Cost-utility analysis (QALY)	60 year old patient with massive rotator cuff tear (without glenohumeral osteoarthritis)	RCT	1. RTSA 2. Rotator cuff repair and re-repair for failures 3. Rotator cuff repair and RTSA for failures	"direct societal costs" and "indirect costs" (non-specific details provided) for a patient’s lifetime	2016, past costs appreciated at 3%, future costs discounted at 3%, USD	RTSA v cuff repair (and possible re-repair): $42,688 v $20,084 RTSA v cuff repair (and possible conversion to RTSA): $42,688 v $20,596	RTSA v cuff repair (and possible re-repair): 12.17 v 12.44 RTSA v cuff repair (and possible conversion to RTSA): 12.17 v 12.55	RTSA v cuff repair (and possible re-repair):-$83,719 (dominated) RTSA v cuff repair (and possible conversion to RTSA): -$58,139 (Dominated); Cuff repair and conversion to RTSA was most cost-effective strategy
Gabig et al. (2023), USA [23]	Retrospective analysis of institutional data	Cost effectiveness analysis (natural units, PROMS)	Patients undergoing RTSA or capsular reconstruction for massive rotator cuff tear without glenohumeral arthritis between 2014—2019 at a single institution, n = 156	RCT	1. RTSA (n = 30) 2. capsular reconstruction (n = 126)	Hospital costs with minimum 1 year follow up of outcomes	No single year of pricing was noted, discounting not stated, USD	RTSA v capsular reconstruction: $18,243 v $14,252 (p =.06)	RTSA v capsular reconstruction: change in ASES 42 v 37 (p =.6)	$798; RTSA similar costs and outcomes
Kang et al. (2017), USA [27]	Modelled	Cost-utility analysis (QALY)	70 year old female patients with massive irreparable rotator cuff tear (RCA or glenohumeral arthritis not mentioned)	RCT	1. RTSA 2. HA 3. Arthroscopic debridement with biceps tenotomy (AB-BT) 4. Physical Therapy	Health system payer perspective (Medicare reimbursement) for a patient's lifetime	2011, 3% annual discount rate, USD	1. RTSA v Physical Therapy: $33,147 v $10,014 2. RTSA v HA: $33,147 v $24,287 3. RTSA v AB-BT: $33,147 v $7,675	1. RTSA v Physical Therapy: 7.7 v 7.0 2. RTSA v HA: 7.7 v 7.35 3. RTSA v AB-BT: 7.7 v 6.7	RTSA v Physical Therapy: $33,048 RTSA v HA: $25,315 RTSA v AB-BT: $25,472; RTSA cost effective
Makhni et al. (2016), USA [29]	Modelled	Cost-utility analysis (QALY)	65 year old patient with massive rotator cuff tear (without cuff-tear arthropathy)	RCT	1. RTSA 2. Rotator cuff repair 3. Nonoperative care	"societal perspective" (non-specific details provided) for a patient's lifetime	2014, adjusted for CPI, utility discount 10%, USD	1. RTSA v cuff repair: $48,316 v $28,732 2. RTSA v nonoperative: $48,316 v $14,559	1. RTSA v cuff repair: 11.72 v 11.73 2. RTSA v nonoperative: 11.72 v 11.02	RTSA v cuff repair: -$1,958,399 (Dominated) RTSA v nonoperative: $48,224; RTSA had higher costs and similar outcomes compared to cuff repair^e^
Oeding et al. (2025), USA [38]	Modelled	Cost-utility analysis (QALY)	Patients with massive irreparable rotator cuff tear without glenohumeral arthropathy	RCT	1. RTSA 2. Capsular reconstruction 3. Trapezius tendon transfer 4. Subacromial balloon spacer	Payer perspective (commercial or government) over 10 years	No single year of pricing was noted, 3% annual discount rate for costs and outcomes, USD	RTSA v capsular reconstruction: $26,896 v $30,540 RTSA v trapezius tendon transfer: $26,896 v $25,819 RTSA v subacromial balloon spacer: $26,896 v $16,412	RTSA v capsular reconstruction: 3.78 v 6.17 RTSA v trapezius tendon transfer: 3.78 v 5.33 RTSA v subacromial balloon spacer: 3.78 v 5.59	RTSA v capsular reconstruction: $1,525 RTSA v trapezius tendon transfer: -$695 (Dominated) RTSA v subacromial balloon spacer: -$5,792 (Dominated); Capsular reconstruction cost-effective compared to RTSA which was dominated by tendon transfer and balloon spacer^f^

Abbreviations: *AB-BT *Arthroscopic debridement with biceps tenotomy, *ASES *American Shoulder and Elbow Surgeons score, *ATSA* anatomical total shoulder arthroplasty, *AVN* avascular necrosis, *GBP* British pound sterling, *HA* Hemi Arthroplasty, *ICER* Incremental Cost Effectiveness Ratio, *MCID* Minimum Clinically Important Difference, *OA *Osteoarthritis,* ORIF* Open Reduction Internal Fixation, *PROMS *Patient Reported Outcome Measures, *PVI* Procedure Value Index, *QALY *Quality Adjusted Life Year, *R&R* Ream-and-Run Arthroplasty, *RCA* Rotator Cuff Arthropathy, *RCT *Rotator Cuff Tear, *RTSA* Reverse Total Shoulder Arthroplasty, *SANE* Single Assessment Numeric Evaluation, *SST* Simple Shoulder Test, *TSA* Total Shoulder Arthroplasty, *USD* US Dollar, *VAS* Visual Analogue Scale

a – for the hypothetical nonoperative group, preoperative index values and costs were assumed by original authors to continue unchanged in the two postoperative years and are reflected in our ICER calculations, using costs and index values from Table II, Page 2003

b – RTSA was cost-effective for individuals 67 years of age or younger, and ATSA was the dominant strategy for patients 77 years or older

c – study authors concluded that RTSA was cost-effective (based on an ICER of $94,118/QALY in 2012)

d – sensitivity analysis by the study authors did not find a significant difference in cost or outcomes between the groups

e – primary RTSA became a cost-effective strategy only if primary cuff repair had high retear rates and progressionto cuff tear arthropathy

f – study authors concluded that capsular reconstruction was most cost-effective option

### Quality assessment

Study quality was assessed with the 10-point, 33-item Drummond checklist, a commonly used tool in health economics [14]. Two reviewers (MLC, SMS) independently scored studies; conflicts were resolved by consensus. Each criterion was scored as 1 (‘yes’), 0.5 (‘cannot tell’), or 0 (‘no’) [11]. Total scores classified quality as: 9–10 = ‘good’, 6–8.5 = ‘fair’, and ≤ 5.5 = ‘poor’.

### Data analysis

Meta-analysis of economic evaluations is rarely feasible due to study heterogeneity [15], which was also true of the current study. Study characteristics were too varied to enable equitable comparisons. Outcomes were assessed with various tools and at different time periods. Included costs varied between studies and were often partially described. Model-based analyses used varied assumptions as data were extrapolated beyond the observed evidence. Given study heterogeneity a narrative synthesis of evidence was conducted per Synthesis Without Meta-analysis (SWiM) guidelines [16]. The threshold for considering an intervention cost-effective is debated [17]. Historically, $50,000 per QALY was a commonly referenced threshold [17]. Recently, $100,000 to $150,000 per QALY is more commonly used in published economic analyses, which Neumann and Kim suggested had theoretical and empirical support [17]. For the current study, interventions with an ICER < US$100,000 per QALY were considered cost-effective relative to comparators. When ICERs were missing or unclear, they were calculated from reported costs and effects.

## Results

### Included studies

**Fig. 1:**
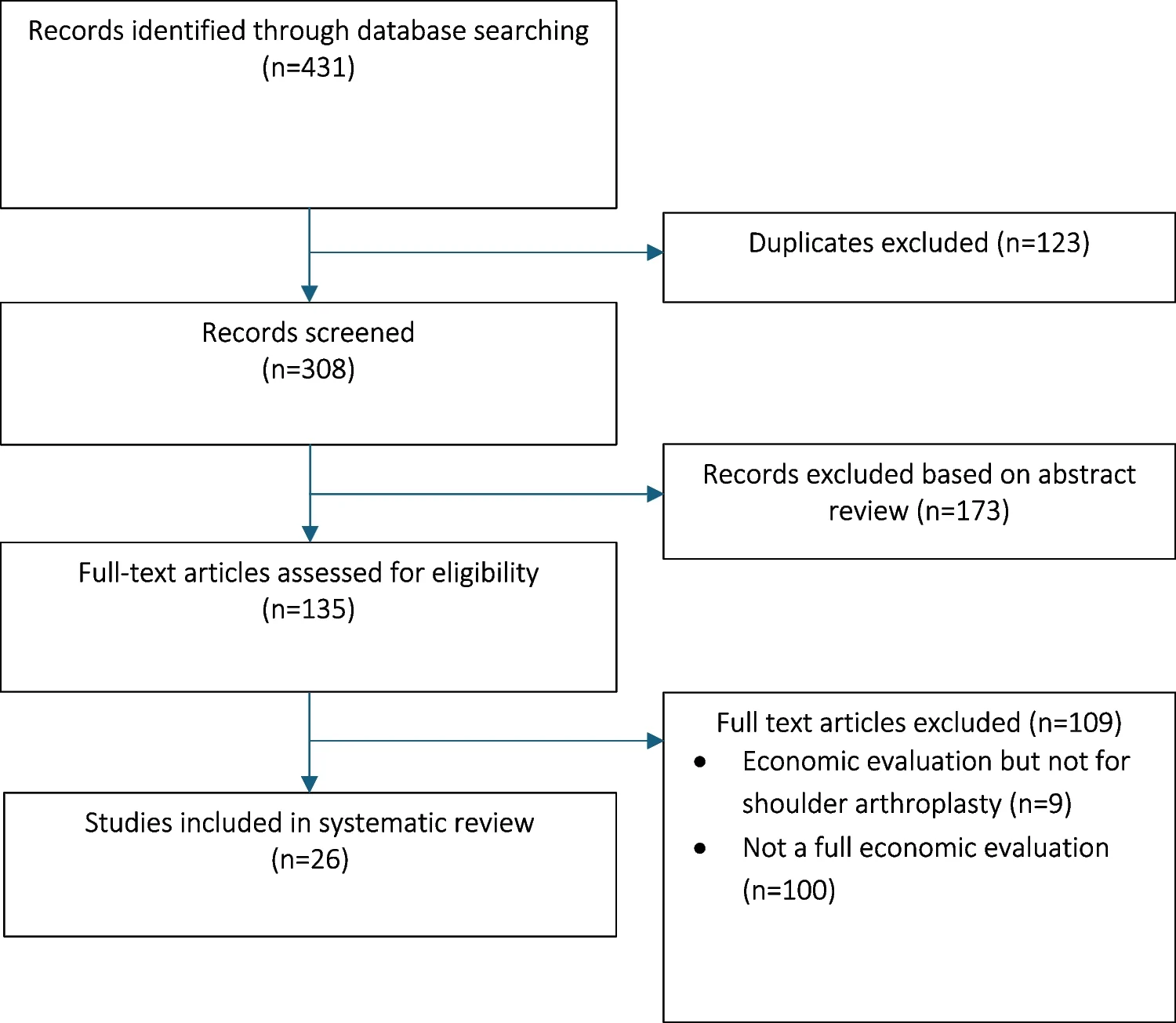
Study identification and selection

From 308 potentially eligible studies, 135 full-text articles were reviewed, and 26 studies met the inclusion criteria (Fig. 1).

### Study characteristics

Study details are provided in Table 1. A pictorial summary of the key results is provided in Table 2..

**Table 2:** Pictorial summary of results

Comparison	Surgical indication	Study	Cost (less expensive)	Clinical effectiveness (more effective)	Conclusion (cost-effective)
	OA with intact cuff	Oeding et al. (2024) [18]		≈	when patient > 67 years
		Polisetty et al. (2021) [19]	≈	≈	≈
		Valsamis et al. (2024) [5]	≈	≈	≈
	Arthritis (mostly OA)	Bhat et al. (2016) [25]			
		Davies et al. (2025) [26]	when patient > 60 years		
		Lapner et al. (2021) [24]			
		Mather et al. (2010) [23]			
	Proximal humerus fracture	Abdel Khalik et al. (2022) [32]			
		Austin et al. (2020) [31]			
		Bjordal et al. (2022) [33]			
	Proximal humerus fracture	Abdel Khalik et al. (2022) [32]			
		Nwachukwu et al. (2016) [35]			
		Osterhoff et al. (2017) [36]			
		Sheibani-Rad et al. (2021) [34]			
	Massive rotator cuff tear	Castagna et al. (2019) [39]			
		Dornan et al. (2017) [38]			
		Makhni et al. (2016) [37]		≈	
	Massive rotator cuff tear	Castagna et al. (2019) [39]			
		Kang et al. (2017) [41]			
		Makhni et al. (2016) [37]			

*NB* includes comparisons with three or more studies

anatomical total shoulder arthroplasty;  reverse total shoulder arthroplasty;  hemi-arthroplasty;  open reduction internal fixation;  rotator cuff repair;  non-operative management

### Cost-effectiveness

#### Arthritis, rotator cuff arthropathy and mixed diagnoses

1. ATSA versus RTSA for OA or mixed diagnoses.

Three studies, including 7439 patients, compared ATSA with RTSA for glenohumeral OA with intact rotator cuff [5, 18, 19]. Oeding et al. compared ATSA with RTSA over a lifetime using a 72-year-old reference case [18]. ATSA was marginally less expensive ($29,077 vs $31,199) and similarly effective (QALY 9.43 vs 9.44); RTSA was not a cost-effective alternative (ICER $212,200). The results were sensitive to patient age with RTSA cost-effective in those aged ≤ 67 years, whereas ATSA was dominant in those aged ≥ 77 years. Valsamis et al. compared ATSA with RTSA for patients ≥ 60 years using UK National Joint Registry data. [5] Although the ICER indicated that ATSA was dominant, no statistically significant or clinically important differences were found for costs or outcomes, suggesting equivalence. Polisetty et al. calculated a procedure value index (patient-reported outcomes expressed in units of minimum clinically important difference (MCID)/mean cost). [19] Like Valsamis et al., no statistically significant differences in costs or outcomes between RTSA and ATSA were identified from hospital, patient, and third-party payer perspectives.

Two additional studies also compared ATSA with RTSA (including 794 patients), however, the preoperative joint pathology was different between the compared groups. Berglund et al. reported similar clinical improvements but slightly lower hospital costs for ATSA ($13,281 vs $14,865, p < 0.001), leading to favourable procedure value indices for ATSA for most measures (p < 0.05) [20]. However, groups differed at baseline: 95.9% of ATSA patients had OA and an intact rotator cuff, whereas 87.9% of RTSA patients had OA with cuff tear. Flinkkila et al. compared ATSA for OA to RTSA for cuff arthropathy using Finnish QALY and costs data; ATSA had greater QALYs (0.54 vs 0.32) and lower costs ($14,138 vs $15,616), indicating ATSA dominance [21]. Once again, different indications for surgery between groups prevents fair outcome/cost comparisons.

2. ATSA or RTSA versus non-operative care for mixed diagnoses.

One study compared 150 patients (46% OA, 40% cuff arthropathy) undergoing ATSA or RTSA with a “hypothetical” non-operative care group using a pre-post design [22]. Costs came from insurer claims one year preoperatively, and one and two years postoperatively; productivity losses were patient-reported via a questionnaire. Surgery was more expensive and more effective for non-workers (ICER $60,770/QALY). For working-age patients, surgery improved productivity, rendering TSA dominant with lower total costs and more QALYs.

3. ATSA versus HA for glenohumeral arthritis.

Four modelled studies compared ATSA with HA for arthritis (mostly OA), finding ATSA was dominant or cost-effective from 30 months [23] to a lifetime horizon [24]. Lapner et al. used utility values for HA for fracture, not arthritis, due to inadequate arthritis data. Findings were robust to changes in most variables and assumptions from a Canadian publicly funded healthcare system perspective. [24] Mather et al. used hip arthroplasty utility values, not shoulder values, finding that ATSA was dominant over HA, which remained the dominant strategy in sensitivity analyses until the utility of ATSA equalled HA. [23] Bhat et al. [25] and Mather et al. [23] claimed societal perspectives; however, costs were estimated from Medicare reimbursement data only, which Bhat et al. indicated “did not include implant costs, other direct costs, or indirect costs to the provider or hospital system” (pg 2491). [25] Davies et al. provided ICERs by age and gender using hospital costs and EQ-5D scores mapped from Oxford Shoulder Scores. [26] Propensity score matching was used to reduce baseline bias. ATSA was dominant for patients aged ≤ 60 years, reflecting higher HA revision rates. For patients older than 60 years, ATSA was cost-effective (ICERs ranged from $185 to $1,837/QALY).

4. RTSA versus HA for cuff arthropathy and mixed diagnoses.

Two US studies compared RTSA with HA; RTSA was consistently more expensive and variably effective. Chawla et al. retrospectively analysed 72 patients with mixed diagnoses (74% OA) up to two years, finding RTSA more costly with similar effectiveness; baseline differences may have confounded results [27]. Coe et al.’s modelled lifetime costs and outcomes for 70-year-olds with rotator cuff arthropathy found RTSA was not cost-effective (ICER $134,331/QALY) [28]. Utilities were calculated from the SF-6D, a generic quality of life measure, which might lack sensitivity for measuring outcomes following shoulder surgery.

5. RTSA press-fit versus RTSA cemented for mixed diagnoses.

Berglund et al. compared press-fit to cemented RTSA for mostly cuff arthropathy (87%). [29] Retrospective analysis of 176 patients with ≥ two year follow-up found lower hospital costs for press-fit (p < 0.05), similar Simple Shoulder Test scores (p > 0.05), leading to a superior procedure value index for press-fit (1.51 vs 1.03, p < 0.001).

6. ATSA versus ream-and-run arthroplasty.

Chawla et al. retrospectively compared 433 patients with OA (74%) or rotator cuff arthropathy (13%). [30] At two years, ream-and-run arthroplasty had lower hospital costs ($10,637 vs $12,717, p < 0.001) and similar QALYs (1.19 vs 1.05, p = 0.361).

#### Proximal humerus fractures

1. RTSA versus ORIF for proximal humerus fractures.

Austin et al.’s lifetime model found RTSA was more expensive but more effective, with ICERs of $4,462/QALY and $30,991/QALY from Medicare and hospital perspectives, respectively [31]. The authors could not identify published utility values for RTSA in the literature and therefore substituted HA utility values. Abdel Khalik et al. also found RTSA was cost-effective versus ORIF (ICER $1,253/QALY) in Canada [32]. A Norwegian within-trial evaluation (n = 124) found RTSA had higher costs and lower QALYs than ORIF over two years, such that ORIF was dominant; however, the authors reported that the differences between groups for costs and QALYs were not statistically significant [33].

2. RTSA versus HA for proximal humerus fractures.

Four US or Canadian modelled studies found RTSA to be more effective than HA, although costs varied. One US study found RTSA less costly, which the authors suggested might have been due to lower procedural and follow-up costs for RTSA compared to other studies, leading to RTSA dominance [34]. Nwachukwu et al. reported that RTSA had higher costs, with ICERs ranging from $6,864 to$70,109 per QALY from Medicare and hospital cost perspectives [35]. Canadian studies found RTSA costlier but cost-effective (ICERs $1,616–$14,409/QALY) [32, 36].

3. RTSA versus non-operative care for proximal humerus fractures.

Two modelled studies found RTSA to be more costly and effective than non-operative care, with ICERs $7,377–$54,174/QALY [32, 35]. Nwachukwu et al. assumed that the cost of non-operative management was zero, which likely underestimates the cost-effectiveness of RTSA.[35]

#### Massive rotator cuff tears (excluding rotator cuff arthropathy)

1. RTSA versus a surgical alternative for massive rotator cuff tears.

Five modelled studies compared RTSA for massive rotator cuff tear with one or more surgical alternatives. In Makhni et al.’s study, RTSA was dominated by rotator cuff repair (more expensive and marginally poorer outcomes) provided that progression from cuff retear to end-stage cuff arthropathy was less than 89% over a patient’s lifetime. When RTSA was required following failed cuff repair that progressed to end-stage CTA, primary RTSA became a cost-effective strategy. [37] Dornan et al. compared three surgical pathways over a patient’s lifetime and found that both cuff repair with possible conversion to RTSA following repair failure and cuff repair with possible re-repair were dominant (less costly and more effective) compared to primary RTSA. [38] However, primary RTSA was cost-effective when the utility of RTSA exceeded that of cuff repair by at least 0.04 QALYs per year. Although Dornan et al. [38] and Makhni et al. [37] reported using a “societal perspective”, few details were provided regarding which costs were included. Castagna’s Italian study used direct medical costs for procedures, implants and hospital care and found that RTSA was dominated (more costly and less effective) by a subacromial spacer or cuff repair at 24 months [39]. Oeding et al. (2025)found that RTSA was the least effective operation, providing the fewest QALYs gained, although the method used to convert Constant-Murley scores to QALYs was unclear [40]. RTSA was dominated, with superior capsular reconstruction being the preferred strategy. Kang et al. found RTSA was the most effective treatment option when compared with HA (ICER $25,315/QALY), or arthroscopic debridement with biceps tenotomy ($25,472/QALY) [41]. One retrospective analysis of a single institution’s cost and outcome data compared RTSA with capsular reconstruction for massive rotator cuff tears (n = 156) [42]. Only direct costs incurred by the hospital for the operation and use of facilities were included. Outcomes were determined using the American Shoulder and Elbow Surgeons score (ASES) at 12 months. RTSA was cost-effective (ICER $798/ASES improvement), though Gabig et al. did not find statistically significant differences between RTSA and reconstruction for costs ($18,243 vs $14,252) or outcomes (change in ASES score: 42 vs 37). Studies by Makhni et al. [37], Dornan et al. [38], Gabig et al. [42], and Oeding et al. [40] assumed no baseline glenohumeral arthritis; however, this important variable was not mentioned by Castagna et al. [39] or Kang et al. [41] In most studies, modelling required major assumptions regarding effectiveness because health-utility states were “rarely reported” (pg 1778) [37] in studies regarding cuff tears.

2. RTSA versus nonoperative care for massive rotator cuff tears.

Three modelled studies compared RTSA with nonoperative care for massive rotator cuff tears. In Italy, Castagna et al. found RTSA nearly twice the cost and less effective than non-operative care at 24 months [39]. In the USA, Makhni et al. found RTSA costlier and more effective than non-operative care at 30 months (ICER $48,224/QALY) [37]. Kang et al., also in the USA, found RTSA more effective but three times more expensive than physical therapy (ICER $33,048/QALY); sensitivity analysis suggested physical therapy could be cost-effective with a slight increase in utility [41].

## Discussion

This systematic review investigated the cost-effectiveness of TSA for glenohumeral arthritis, rotator cuff arthropathy, massive rotator cuff tears and proximal humerus fractures. Due to heterogeneity among the 26 included studies, quantitative data pooling was not possible. Narrative synthesis revealed consistent findings for some comparisons, which were limited to a small number of studies conducted in high-income countries. In summary, the comparisons with the most consistent findings across multiple studies showed that ATSA was dominant or cost-effective compared with HA for glenohumeral OA in four studies. For complex proximal humerus fractures, RTSA was dominant or cost-effective compared with HA in all four studies. For massive rotator cuff tears, primary rotator cuff repair was substantially less costly and marginally more effective than primary RTSA in all three studies. For ATSA versus RTSA for OA and intact cuff, procedures were similarly effective, and costs were similar or less for ATSA in all three studies. Evidence was inconsistent or insufficient for comparing ATSA or RTSA with nonoperative care for OA, RTSA with HA for rotator cuff arthropathy, and RTSA with ORIF for proximal humerus fractures.

ATSA dominated or was cost-effective over HA for primary glenohumeral OA in four studies of good or fair quality conducted in the USA, Canada, and the UK [23]. A 2021 meta-analysis determined that ATSA was more effective than HA for range of movement, pain, daily function, and revision [43]. Although HA has been suggested for younger patients [43], Davies et al. and Bhat et al. found ATSA to be less expensive and more effective when considered over a patient’s lifetime.[25]

The relative superiority of ATSA compared to RTSA for OA with an intact rotator cuff remains debated. In the current review, ATSA was comparable or cost-effective versus RTSA, but results are primarily limited to three studies, ranging from poor to good quality, with follow-up time ranging from two years to a patient’s lifetime. A recent meta-analysis of 12 studies found RTSA superior for pain, function and complications; however all studies were non-randomised retrospective studies, which precludes unbiased comparisons between groups [44]. Cost-effectiveness appears to vary according to patient age. Oeding et al. found RTSA was cost-effective for patients 67 years or younger, and ATSA was cost-effective or dominant for patients older than this [18]. High quality, prospective RCTs of comparable groups are clearly necessary to robustly determine the cost-effectiveness of RTSA and ATSA in OA with intact cuff; several such studies are currently underway [45, 46].

RTSA’s main clinical advantage is reported in patients with deficient rotator cuff tendons [5]. However, this perceived clinical advantage did not translate into RTSA being a cost-effective treatment in two studies investigating cuff arthropathy, and results were mixed for massive cuff tears. One retrospective study found HA to be dominant, being marginally more effective and less costly than RTSA for patients with cuff arthropathy, though the comparison was limited to two years of follow-up and the extent of preoperative glenoid erosion was not described in either group [27]. The second modelled study reported RTSA was cost-effective compared to HA (ICER $94,118/QALY in 2012), however, when recalculated in 2024, RTSA was no longer below the $100,000/QALY willingness-to-pay threshold (ICER $134,331/QALY) [28].

For massive cuff tears, primary repair was the dominant strategy over RTSA in all three studies; it was substantially cheaper, although differences in effects were small and arguably not clinically important, and varied in the sensitivity analyses [37]. Most studies required major assumptions, such as assuming utility values were equal after cuff repair and RTSA [38] because utility values were “rarely reported”(p. 1778) [37]. Prospective studies or registries with robust utility data would improve future economic evaluations.

Regarding proximal humerus fractures, RTSA was either cost-effective or the dominant strategy in five of six studies. A systematic review of six studies comparing RTSA with ORIF reported similar pain and function scores between the procedures and mixed results for range of movement; ORIF led to more revision surgery, but RTSA was associated with a higher overall complication rate [47]. HA has been the traditional treatment option for complex proximal humerus fractures not amenable to internal fixation [35, 48]. In the current review, RTSA was more effective than HA in all four studies, which contrasts with findings from a systematic review of 14 studies, which found similar patient-reported and range of motion outcomes between the two groups, but four times greater odds of complications over a mean follow-up time of 43.5 months [49]. The modelled studies in our review reported lifetime effectiveness favouring RTSA and lifetime ICERs below the $100,000/QALY willingness-to-pay threshold, suggesting that RTSA is cost-effective compared with HA.

The current study builds on Cregar et al.’s review which included nine studies based in the USA. [9] Some of these studies were excluded from our review; Renfree et al. [50] did not include a comparison group, and Bachman et al. [51] compared RTSA with hip arthroplasty. The additional 19 studies included in our review add an international perspective from nine non-USA studies. The most notable addition is the inclusion of five studies addressing proximal humerus fractures.

Our conclusions are limited by the type and quality of the included studies. Modelled studies rely on assumptions derived from limited published evidence and extrapolate costs and outcomes beyond the observed data. For example, some studies assumed effectiveness data from other procedures, such as hip arthroplasty. Of the 11 studies using real data, ten studies were of non-randomised design, and baseline comparability between treatment groups was therefore not assured, and groups sometimes had overtly different preoperative pathology (e.g., osteoarthritis versus cuff-tear arthropathy); differences in postoperative outcomes cannot be confidently attributed to the interventions themselves. For the one RCT with cost-effectiveness analysis, the relatively small sample and two-year follow-up constrain comparisons and conclusions regarding lifetime cost-effectiveness [33]. TSA is a technically demanding procedure[41], and outcomes for one surgeon or site might differ from another, limiting the generalisability of the studies. Costs were commonly considered from the healthcare perspective, often based on USA Medicare reimbursement data [3], and broader societal costs were not included, such as lost productivity. Costs for goods and services can vary substantially between settings, making comparisons difficult. None of the included studies were in low-income or middle-income countries and the generalisability of the findings to lower resource settings is unknown. We are confident we have retrieved the key published economic evaluations on TSA; however, in addition to the limitations within each study, there are also limitations to our review. Most notably, we limited our search to English language peer-reviewed literature and did not search the grey literature or publish the protocol for this review.

## Conclusions

While TSA appears to offer value for money for certain shoulder indications, large and high-quality, randomised, prospective economic evaluations that include both societal and healthcare provider perspectives, with equitable baseline characteristics between groups are needed to provide rigorous evidence to guide value-based care in shoulder arthroplasty. Cost-effectiveness varies by cultural and economic context, so evaluations are needed beyond high-income countries.

## Supplementary Information

Below is the link to the electronic supplementary material.

Supplementary file1 (DOCX 26 KB)

Supplementary file2 (DOCX 46.1 KB)

## Data Availability

No datasets were generated or analysed during the current study.
