# Supplementary material for: Cost-effectiveness of total shoulder arthroplasty: A systematic review of the evidence
Source: Int Orthop. 2026 Jun 29;50(9):2395–414. doi: 10.1007/s00264-026-06830-2 (PMC13562116; doi:10.1007/s00264-026-06830-2)
Supplement: Supplementary file 1 [file 264_2026_6830_MOESM1_ESM.docx]

**Supplementary Table 1. Summary of database searches and results**

| **Database Platform** | | **Embase** | |
| --- | --- | --- | --- |
| Database | | Embase | |
| Date of Search | | July 28, 2025 | |
| Limiters | | English only; Humans Only | |
| **#** | **Search Terms** | | **Results** |
| 1 | “Total Shoulder Replacement*” OR “Total Shoulder Arthroplast*” OR “Shoulder Replacement Arthroplast*” OR “Shoulder Replacement*” OR “Reverse Total Shoulder Arthroplast*” OR “Shoulder Hemiarthroplast*” OR “Shoulder Arthroplast*” OR “Reverse Shoulder Arthroplast*” OR “Shoulder Joint Surger*” OR “Shoulder Joint*” OR “Shoulder Joint Replacement*” OR “Glenohumeral Arthroplast*” | | 17,845 |
| 2 | "Cost-Benefit Analys?s" OR "Economic Evaluation*" OR "Costs and  Cost Analysis" OR "Cost Analys?s" OR "Return on Investment" OR "Return to Investment" OR "Cost Effectiveness Analys?s" OR "Cost  Utility Analys?s" OR “Cost Effectiveness” OR “Cost Efficiency  Analys?s” OR “Cost of Illness*” OR “Cost Minimi?ation Analys?s” | | 292,717 |
|  | 1 AND 2  *Total Number of Literature Found When Combined* | |  |
|  |  |  | 265 |

| **Database Platform** | | **EBSCO** | |
| --- | --- | --- | --- |
| Database | | CINAHL Complete | |
| Date of Search | | July 28, 2025 | |
| Limiters | | English only; Humans Only | |
| **#** | **Search Terms** | | **Results** |
| 1 | “Total Shoulder Replacement*” OR “Total Shoulder Arthroplast*” OR “Shoulder Replacement Arthroplast*” OR “Shoulder Replacement*” OR “Reverse Total Shoulder Arthroplast*” OR “Shoulder Hemiarthroplast*” OR “Shoulder Arthroplast*” OR “Reverse Shoulder Arthroplast*” OR “Shoulder Joint Surger*” OR “Shoulder Joint*” OR “Shoulder Joint Replacement*” OR “Glenohumeral Arthroplast*” | | 2,755 |
| 2 | "Cost-Benefit Analys?s" OR "Economic Evaluation*" OR "Costs and  Cost Analysis" OR "Cost Analys?s" OR "Return on Investment" OR "Return to Investment" OR "Cost Effectiveness Analys?s" OR "Cost  Utility Analys?s" OR “Cost Effectiveness” OR “Cost Efficiency  Analys?s” OR “Cost of Illness*” OR “Cost Minimi?ation Analys?s” | | 41,808 |
|  | 1 AND 2  *Total Number of Literature Found When Combined* | |  |
|  |  |  | 50 |

| **Database Platform** | | **EBSCO** | |
| --- | --- | --- | --- |
| Database | | Medline Complete | |
| Date of Search | | July 28, 2025 | |
| Limiters | | English only; Humans Only | |
| **#** | **Search Terms** | | **Results** |
| 1 | “Total Shoulder Replacement*” OR “Total Shoulder Arthroplast*” OR “Shoulder Replacement Arthroplast*” OR “Shoulder Replacement*” OR “Reverse Total Shoulder Arthroplast*” OR “Shoulder Hemiarthroplast*” OR “Shoulder Arthroplast*” OR “Reverse Shoulder Arthroplast*” OR “Shoulder Joint Surger*” OR “Shoulder Joint*” OR “Shoulder Joint Replacement*” OR “Glenohumeral Arthroplast*” | | 5,995 |
| 2 | "Cost-Benefit Analys?s" OR "Economic Evaluation*" OR "Costs and  Cost Analysis" OR "Cost Analys?s" OR "Return on Investment" OR "Return to Investment" OR "Cost Effectiveness Analys?s" OR "Cost  Utility Analys?s" OR “Cost Effectiveness” OR “Cost Efficiency  Analys?s” OR “Cost of Illness*” OR “Cost Minimi?ation Analys?s” | | 192,347 |
|  | 1 AND 2  *Total Number of Literature Found When Combined* | |  |
|  |  |  | 116 |
|  |  |  |  |
|  |  | |  |
