# Supplementary material for: Cost-effectiveness of total shoulder arthroplasty: A systematic review of the evidence
Source: Int Orthop. 2026 Jun 29;50(9):2395–414. doi: 10.1007/s00264-026-06830-2 (PMC13562116; doi:10.1007/s00264-026-06830-2)
Supplement: Supplementary file 2 [file 264_2026_6830_MOESM2_ESM.docx]

**Supplementary Table 2a. Detailed quality assessment questions with sub-questions from the Drummond checklist: Osteoarthritis, rotator cuff arthropathy or mixed diagnosis**

|  | **Mather et al (2010)** | **Bhat et al (2016)** | **Lapner et al (2021)** | **Davies et al (2025)** | **Berglund et al (A) (2019)** | **Polisetty et al (2021)** | **Oeding et al (A) (2024)** | **Flinkkila et al (2024)** | **Valsamis et al (2024)** | **Chawla et al (2021)** | **Grobet et al (2021)** | **Coe et al (2012)** | **Chawla et al (2022)** | **Berglund et al (B) (2019)** |
| --- | --- | --- | --- | --- | --- | --- | --- | --- | --- | --- | --- | --- | --- | --- |
| **1. Was a well-defined question posed in answerable form?** | | | | | | | | | | | | | | |
| 1.1 Did the study examine both costs and effects of the service(s) or programme(s) over an appropriate time horizon? | Yes | Yes | Yes | Yes | Yes | Yes | Yes | Yes | Yes | Yes | Yes | Yes | Yes | Yes |
| 1.2 Did the study involve a comparison of alternatives? | Yes | Yes | Yes | Yes | Yes | Yes | Yes | Yes | Yes | Yes | Yes | Yes | Yes | Yes |
| 1.3 Was a perspective for the analysis stated and was the study placed in any particular decision-making context? | Yes | Yes | Yes | Yes | No | Yes | No | Yes | Yes | No | Yes | Yes | No | No |
| 1.4 Were the patient population and any relevant subgroups adequately defined? | Yes | Yes | Yes | Yes | Yes | Yes | Yes | Yes | Yes | Yes | Yes | Yes | Yes | Yes |
| **2. Was a comprehensive description of the competing alternatives given? (i.e. can you tell who did what to whom, where, and how often?)** | | | | | | | | | | | | | | |
| 2.1 Were any relevant alternatives omitted? | Yes | No | No | No | No | No | No | No | No | No | No | No | No | No |
| 2.2 Was (should) a ‘do nothing’ alternative (be) considered? | No | Yes | No | No | No | No | No | No | No | No | Yes | No | No | No |
| 2.3 Were relevant alternatives identified for the patient subgroups? | No | No | No | No | No | No | No | No | No | No | No | No | No | No |
| **3. Was the effectiveness of the programmes or services established?** | | | | | | | | | | | | | | |
| 3.1 Was this done through a randomized controlled clinical trial? If so, did the trial protocol reflect what would happen in regular practice? | No | No | No | No | No | No | No | No | No | No | No | No | No | No |
| 3.2 Were effectiveness data collected and summarized through a systematic overview of clinical studies? If so, were the search strategy and rules for inclusion or exclusion outlined? | Yes | Yes | No | No | No | No | Yes | No | No | No | No | No | No | No |
| 3.3 Were observational data or assumptions used to establish effectiveness? If so, were any potential biases recognized? | No | No | Yes | Yes | Yes | Yes | Yes | Yes | Yes | Yes | Yes | Yes | Yes | Yes |
| **4. Were all the important and relevant costs and consequences for each alternative identified?** | | | | | | | | | | | | | | |
| 4.1 Was the range wide enough for the research question at hand? | Yes | Yes | Yes | Yes | Yes | Yes | Yes | Yes | Yes | No | Yes | Yes | No | Yes |
| 4.2 Did it cover all relevant perspectives? (Possible perspectives include those of patients and third-party payers; other perspectives may also be relevant depending on the particular analysis.) | Yes | No | Yes | No | No | Yes | No | No | No | No | Yes | Yes | No | No |
| 4.3 Were capital costs, as well as operating costs, included? | Can't tell | Can't tell | Can't tell | Can't tell | Can't tell | Can't tell | Can't tell | Can't tell | Can't tell | Can't tell | Can't tell | Can't tell | Can't tell | Can't tell |
| **5. Were costs and consequences measured accurately in appropriate physical units prior to valuation (e.g. hours of nursing time, number of physician visits, lost work-days, gained life-years)?** | | | | | | | | | | | | | | |
| 5.1 Were the sources of resource utilization described and justified? | Yes | Yes | Yes | Yes | Yes | Yes | Yes | Yes | Yes | Yes | Yes | Yes | Yes | Yes |
| 5.2 Were any of the identified items omitted from measurement? If so, does this mean that they carried no weight in the subsequent analysis? | No | No | No | No | No | No | No | No | No | Can't tell | No | No | Can't tell | No |
| 5.3 Were there any special circumstances (e.g. joint use of resources) that made measurement difficult? Were these circumstances handled appropriately? | No | No | No | Yes | No | No | No | Can't tell | Can't tell | No | No | No | No | No |
| **6. Were costs and consequences valued credibly?** | | | | | | | | | | | | | | |
| 6.1 Were the sources of all values clearly identified? (Possible sources include market values, patient or client preferences and views, policymakers’ views, and health professionals’ judgements.) | Yes | Yes | Can't tell | Yes | Yes | Yes | Yes | Yes | Yes | Yes | Yes | Yes | Yes | Yes |
| 6.2 Were market values employed for changes involving resources gained or depleted? | Can't tell | Yes | Yes | Yes | Yes | Yes | Yes | Yes | Yes | Yes | Yes | Can't tell | Yes | Yes |
| 6.3 Where market values were absent (e.g. volunteer labour), or market values did not reflect actual values (e.g. clinic space donated at a reduced rate), were adjustments made to approximate market values? | Can't tell | Can't tell | Can't tell | Yes | Can't tell | Can't tell | Can't tell | No | No | Can't tell | Can't tell | Can't tell | Can't tell | Can't tell |
| 6.4 Was the valuation of consequences appropriate for the question posed (i.e. has the appropriate type or types of analysis—cost-effectiveness, cost–benefit—been selected)? | Yes | Yes | Yes | Yes | Yes | Can't tell | Yes | Yes | Yes | Yes | Yes | Yes | Can't tell | Yes |
| **7. Were costs and consequences adjusted for differential timing?** | | | | | | | | | | | |  |  |  |
| 7.1 Were costs and consequences that occur in the future ‘discounted’ to their present values? | Yes | No | Yes | Yes | No | No | Yes | No | Yes | Yes | No | Yes | Yes | No |
| 7.2 Was any justification given for the discount rate(s) used? | No | No | Yes | Yes | No | No | No | No | Yes | No | Yes | No | No | No |
| **8. Was an incremental analysis of costs and consequences of alternatives performed?** | | | | | | | | | | | |  |  |  |
| 8.1 Were the additional (incremental) costs generated by one alternative over another compared to the additional effects, benefits, or utilities generated? | Yes | No | Yes | Yes | No | No | Yes | No | No | No | Yes | Yes | No | No |
| **9. Was uncertainty in the estimates of costs and consequences adequately characterized?** | | | | | | | | | | | | | | |
| 9.1 If patient-level data on costs or consequences were available, were appropriate statistical analyses performed? | Can't tell | Yes | Can't tell | Yes | Yes | Can't tell | No | Can't tell | Yes | Yes | Yes | Can't tell | Can't tell | Can't tell |
| 9.2 If a sensitivity analysis was employed, was justification provided for the form(s) of sensitivity analysis employed and the ranges or distributions of values (for key study parameters)? | Yes | Yes | Yes | Yes | No | No | Yes | Yes | Yes | No | Yes | Yes | No | No |
| 9.3 Were the conclusions of the study sensitive to the uncertainty in the results, as quantified by the statistical and/or sensitivity analysis? | Yes | Yes | Yes | Yes | No | No | Yes | Yes | No | No | Yes | Yes | No | No |
| 9.4 Was heterogeneity in the patient population recognized, for example by presenting study results for relevant subgroups? | No | Yes | Yes | Yes | No | No | Yes | No | No | Yes | Can't tell | No | Can't tell | No |
| **10. Did the presentation and discussion of study results include all issues of concern to users?** | | | | | | | | | | | | | | |
| 10.1 Were the conclusions of the analysis based on some overall index or ratio of costs to consequences (e.g. cost-effectiveness ratio)? If so, was the index interpreted intelligently or in a mechanistic fashion? | Yes | Can't tell | Yes | Yes | Yes | Yes | Yes | Yes | No | No | Yes | Yes | No | No |
| 10.2 Were the results compared with those of others who have investigated the same question? If so, were allowances made for potential differences in study methodology? | No | Yes | Yes | Yes | Yes | Yes | Yes | Yes | Yes | Yes | Yes | Yes | Yes | Can't tell |
| 10.3 Did the study discuss the generalizability of the results to other settings and patient/client groups? | No | Yes | No | Yes | Yes | Yes | Yes | No | No | Yes | No | Yes | Yes | Yes |
| 10.4 Did the study allude to, or take account of, other important factors in the choice or decision under consideration (e.g. distribution of costs and consequences, or relevant ethical issues)? | Yes | No | Yes | Yes | Yes | No | Can't tell | No | Can't tell | Yes | Yes | Yes | Yes | Yes |
| 10.5 Did the study discuss issues of implementation, such as the feasibility of adopting the ‘preferred’ programme given existing financial or other constraints, and whether any freed resources could be redeployed to other worthwhile programmes? | Yes | No | No | No | No | No | Can't tell | No | No | No | No | No | No | No |
| 10.6 Were the implications of uncertainty for decision-making, including the need for future research, explored? | Yes | Yes | No | No | No | No | Can't tell | No | Yes | Yes | Can't tell | Yes | Yes | Yes |
| Judgement | Good | Fair | Good | Good | Fair | Fair | Fair | Fair | Fair | Fair | Fair | Good | Fair | Poor |

**Supplementary Table 2b. Detailed quality assessment questions with sub-questions from the Drummond checklist: Fractured proximal humerus**

|  | **Nwachukwu et al (2016)** | **Osterhoff et al (2017)** | **Austin et al (2020)** | **Sheibani-Rad et al (2021)** | **Bjordal et al (2022)** | **Abdel Khalik et al (2022)** |
| --- | --- | --- | --- | --- | --- | --- |
| **1. Was a well-defined question posed in answerable form?** | | | | | | |
| 1.1 Did the study examine both costs and effects of the service(s) or programme(s) over an appropriate time horizon? | Yes | Yes | Yes | Yes | Yes | Yes |
| 1.2 Did the study involve a comparison of alternatives? | Yes | Yes | Yes | Yes | Yes | Yes |
| 1.3 Was a perspective for the analysis stated and was the study placed in any particular decision-making context? | Yes | Yes | Yes | Yes | Yes | Yes |
| 1.4 Were the patient population and any relevant subgroups adequately defined? | Yes | Yes | Yes | Yes | Yes | Yes |
| **2. Was a comprehensive description of the competing alternatives given? (i.e. can you tell who did what to whom, where, and how often?)** | | | | | | |
| 2.1 Were any relevant alternatives omitted? | No | Yes | No | No | No | No |
| 2.2 Was (should) a ‘do nothing’ alternative (be) considered? | Yes | No | No | No | No | Yes |
| 2.3 Were relevant alternatives identified for the patient subgroups? | No | No | No | No | No | No |
| **3. Was the effectiveness of the programmes or services established?** | | | | | | |
| 3.1 Was this done through a randomized controlled clinical trial? If so, did the trial protocol reflect what would happen in regular practice? | No | No | No | No | Yes | No |
| 3.2 Were effectiveness data collected and summarized through a systematic overview of clinical studies? If so, were the search strategy and rules for inclusion or exclusion outlined? | Yes | Yes | Can't tell | No | No | Yes |
| 3.3 Were observational data or assumptions used to establish effectiveness? If so, were any potential biases recognized? | Yes | Yes | Can't tell | No | No | Yes |
| **4. Were all the important and relevant costs and consequences for each alternative identified?** | | | | | | |
| 4.1 Was the range wide enough for the research question at hand? | Yes | Yes | Yes | Yes | Yes | Yes |
| 4.2 Did it cover all relevant perspectives? (Possible perspectives include those of patients and third-party payers; other perspectives may also be relevant depending on the particular analysis.) | Yes | Yes | Yes | Can't tell | Yes | Yes |
| 4.3 Were capital costs, as well as operating costs, included? | Can't tell | Can't tell | Can't tell | Can't tell | Can't tell | Can't tell |
| **5. Were costs and consequences measured accurately in appropriate physical units prior to valuation (e.g. hours of nursing time, number of physician visits, lost work-days, gained life-years)?** | | | | | | |
| 5.1 Were the sources of resource utilization described and justified? | Yes | Yes | Yes | Can't tell | Yes | Yes |
| 5.2 Were any of the identified items omitted from measurement? If so, does this mean that they carried no weight in the subsequent analysis? | No | No | No | No | Can't tell | No |
| 5.3 Were there any special circumstances (e.g. joint use of resources) that made measurement difficult? Were these circumstances handled appropriately? | No | No | No | No | No | No |
| **6. Were costs and consequences valued credibly?** | | | | | | |
| 6.1 Were the sources of all values clearly identified? (Possible sources include market values, patient or client preferences and views, policymakers’ views, and health professionals’ judgements.) | Yes | Yes | Can't tell | Can't tell | Yes | Yes |
| 6.2 Were market values employed for changes involving resources gained or depleted? | Can't tell | Can't tell | Can't tell | Can't tell | Yes | Yes |
| 6.3 Where market values were absent (e.g. volunteer labour), or market values did not reflect actual values (e.g. clinic space donated at a reduced rate), were adjustments made to approximate market values? | Can't tell | Can't tell | Can't tell | Can't tell | Can't tell | Can't tell |
| 6.4 Was the valuation of consequences appropriate for the question posed (i.e. has the appropriate type or types of analysis—cost-effectiveness, cost–benefit—been selected)? | Yes | Yes | Yes | Yes | Yes | Yes |
| **7. Were costs and consequences adjusted for differential timing?** | | | | | | |
| 7.1 Were costs and consequences that occur in the future ‘discounted’ to their present values? | Yes | Yes | Yes | Yes | Can't tell | Yes |
| 7.2 Was any justification given for the discount rate(s) used? | No | Yes | No | No | No | No |
| **8. Was an incremental analysis of costs and consequences of alternatives performed?** | | | | | | |
| 8.1 Were the additional (incremental) costs generated by one alternative over another compared to the additional effects, benefits, or utilities generated? | Yes | Yes | Yes | Yes | Yes | Yes |
| **9. Was uncertainty in the estimates of costs and consequences adequately characterized?** | | | | | | |
| 9.1 If patient-level data on costs or consequences were available, were appropriate statistical analyses performed? | Can't tell | Can't tell | No | Can't tell | No | Yes |
| 9.2 If a sensitivity analysis was employed, was justification provided for the form(s) of sensitivity analysis employed and the ranges or distributions of values (for key study parameters)? | Yes | Yes | Yes | Yes | Can't tell | Yes |
| 9.3 Were the conclusions of the study sensitive to the uncertainty in the results, as quantified by the statistical and/or sensitivity analysis? | Yes | Yes | Yes | Yes | No | Yes |
| 9.4 Was heterogeneity in the patient population recognized, for example by presenting study results for relevant subgroups? | No | No | Can't tell | Can't tell | No | Can't tell |
| **10. Did the presentation and discussion of study results include all issues of concern to users?** | | | | | | |
| 10.1 Were the conclusions of the analysis based on some overall index or ratio of costs to consequences (e.g. cost-effectiveness ratio)? If so, was the index interpreted intelligently or in a mechanistic fashion? | Yes | Yes | Yes | Yes | Yes | Yes |
| 10.2 Were the results compared with those of others who have investigated the same question? If so, were allowances made for potential differences in study methodology? | Yes | Yes | Yes | Yes | Yes | Yes |
| 10.3 Did the study discuss the generalizability of the results to other settings and patient/client groups? | Yes | Yes | No | No | Yes | Yes |
| 10.4 Did the study allude to, or take account of, other important factors in the choice or decision under consideration (e.g. distribution of costs and consequences, or relevant ethical issues)? | Yes | No | No | No | Yes | Yes |
| 10.5 Did the study discuss issues of implementation, such as the feasibility of adopting the ‘preferred’ programme given existing financial or other constraints, and whether any freed resources could be redeployed to other worthwhile programmes? | No | No | Can't tell | No | No | Yes |
| 10.6 Were the implications of uncertainty for decision-making, including the need for future research, explored? | No | No | Yes | No | Can't tell | Yes |
| Judgement | Good | Good | Fair | Fair | Fair | Good |

**Supplementary Table 2c. Detailed quality assessment questions with sub-questions from the Drummond checklist: Rotator cuff tear**

|  | **Makhni et al (2016)** | **Dornan et al (2017)** | **Kang et al (2017)** | **Castagna et al (2019)** | **Gabig et al (2023)** | **Oeding et al (2025)** |
| --- | --- | --- | --- | --- | --- | --- |
| **1. Was a well-defined question posed in answerable form?** | | | | | | |
| 1.1 Did the study examine both costs and effects of the service(s) or programme(s) over an appropriate time horizon? | Yes | Yes | Yes | Yes | Can't tell | Yes |
| 1.2 Did the study involve a comparison of alternatives? | Yes | Yes | Yes | Yes | Yes | Yes |
| 1.3 Was a perspective for the analysis stated and was the study placed in any particular decision-making context? | Yes | No | No | Yes | No | Yes |
| 1.4 Were the patient population and any relevant subgroups adequately defined? | Yes | Yes | Yes | Yes | Yes | Yes |
| **2. Was a comprehensive description of the competing alternatives given? (i.e. can you tell who did what to whom, where, and how often?)** | | | | | | |
| 2.1 Were any relevant alternatives omitted? | No | No | No | No | Yes | Can't tell |
| 2.2 Was (should) a ‘do nothing’ alternative (be) considered? | Yes | No | Yes | Yes | No | No |
| 2.3 Were relevant alternatives identified for the patient subgroups? | No | No | No | No | No | No |
| **3. Was the effectiveness of the programmes or services established?** | | | | | | |
| 3.1 Was this done through a randomized controlled clinical trial? If so, did the trial protocol reflect what would happen in regular practice? | No | No | No | No | No | No |
| 3.2 Were effectiveness data collected and summarized through a systematic overview of clinical studies? If so, were the search strategy and rules for inclusion or exclusion outlined? | Yes | No | Yes | Yes | No | Yes |
| 3.3 Were observational data or assumptions used to establish effectiveness? If so, were any potential biases recognized? | No | Yes | Yes | Yes | Yes | Can't tell |
| **4. Were all the important and relevant costs and consequences for each alternative identified?** | | | | | | |
| 4.1 Was the range wide enough for the research question at hand? | Yes | Yes | Yes | Yes | Can't tell | Yes |
| 4.2 Did it cover all relevant perspectives? (Possible perspectives include those of patients and third-party payers; other perspectives may also be relevant depending on the particular analysis.) | Yes | Yes | No | Yes | No | No |
| 4.3 Were capital costs, as well as operating costs, included? | Can't tell | Can't tell | Can't tell | Can't tell | No | Can't tell |
| **5. Were costs and consequences measured accurately in appropriate physical units prior to valuation (e.g. hours of nursing time, number of physician visits, lost work-days, gained life-years)?** | | | | | | |
| 5.1 Were the sources of resource utilization described and justified? | Yes | Yes | Yes | Yes | Yes | Yes |
| 5.2 Were any of the identified items omitted from measurement? If so, does this mean that they carried no weight in the subsequent analysis? | No | No | No | No | No | No |
| 5.3 Were there any special circumstances (e.g. joint use of resources) that made measurement difficult? Were these circumstances handled appropriately? | No | No | No | No | Can't tell | No |
| **6. Were costs and consequences valued credibly?** | | | | | | |
| 6.1 Were the sources of all values clearly identified? (Possible sources include market values, patient or client preferences and views, policymakers’ views, and health professionals’ judgements.) | Yes | Can't tell | Yes | Yes | Yes | Yes |
| 6.2 Were market values employed for changes involving resources gained or depleted? | Can't tell | Can't tell | Can't tell | Yes | Yes | Yes |
| 6.3 Where market values were absent (e.g. volunteer labour), or market values did not reflect actual values (e.g. clinic space donated at a reduced rate), were adjustments made to approximate market values? | Can't tell | Can't tell | Can't tell | Yes | No | Can't tell |
| 6.4 Was the valuation of consequences appropriate for the question posed (i.e. has the appropriate type or types of analysis—cost-effectiveness, cost–benefit—been selected)? | Yes | Yes | Yes | Yes | Yes | Yes |
| **7. Were costs and consequences adjusted for differential timing?** | | | | | | |
| 7.1 Were costs and consequences that occur in the future ‘discounted’ to their present values? | No | Yes | Yes | Yes | Yes | Yes |
| 7.2 Was any justification given for the discount rate(s) used? | No | No | No | Yes | Yes | No |
| **8. Was an incremental analysis of costs and consequences of alternatives performed?** | | | | | | |
| 8.1 Were the additional (incremental) costs generated by one alternative over another compared to the additional effects, benefits, or utilities generated? | Yes | Yes | Yes | Yes | Yes | Yes |
| **9. Was uncertainty in the estimates of costs and consequences adequately characterized?** | | | | | | |
| 9.1 If patient-level data on costs or consequences were available, were appropriate statistical analyses performed? | Can't tell | Can't tell | Can't tell | Can't tell | Yes | Yes |
| 9.2 If a sensitivity analysis was employed, was justification provided for the form(s) of sensitivity analysis employed and the ranges or distributions of values (for key study parameters)? | No | Yes | Yes | Yes | No | Yes |
| 9.3 Were the conclusions of the study sensitive to the uncertainty in the results, as quantified by the statistical and/or sensitivity analysis? | Yes | Yes | Yes | Yes | No | Yes |
| 9.4 Was heterogeneity in the patient population recognized, for example by presenting study results for relevant subgroups? | No | No | No | No | No | No |
| **10. Did the presentation and discussion of study results include all issues of concern to users?** | | | | | | |
| 10.1 Were the conclusions of the analysis based on some overall index or ratio of costs to consequences (e.g. cost-effectiveness ratio)? If so, was the index interpreted intelligently or in a mechanistic fashion? | Yes | Yes | Yes | Yes | Yes | Yes |
| 10.2 Were the results compared with those of others who have investigated the same question? If so, were allowances made for potential differences in study methodology? | Yes | Yes | No | Yes | Yes | Yes |
| 10.3 Did the study discuss the generalizability of the results to other settings and patient/client groups? | Yes | Yes | No | Yes | Yes | Yes |
| 10.4 Did the study allude to, or take account of, other important factors in the choice or decision under consideration (e.g. distribution of costs and consequences, or relevant ethical issues)? | No | No | No | Yes | Can't tell | Yes |
| 10.5 Did the study discuss issues of implementation, such as the feasibility of adopting the ‘preferred’ programme given existing financial or other constraints, and whether any freed resources could be redeployed to other worthwhile programmes? | No | No | Yes | Yes | Yes | No |
| 10.6 Were the implications of uncertainty for decision-making, including the need for future research, explored? | No | No | No | Yes | No | Yes |
| Judgement | Fair | Fair | Fair | Good | Fair | Good |
